# Supplementary material for: Therapeutic Potential in Wound Healing of Allogeneic Use of Equine Umbilical Cord Mesenchymal Stem Cells
Source: Int J Mol Sci. 2024 Feb 16;25(4):2350. doi: 10.3390/ijms25042350 (PMC10889822; doi:10.3390/ijms25042350)
Supplement: Supplementary file 1 [file ijms-25-02350-s001.zip › ijms-2825448-supplementary.pdf]

Figure S1: Ultrasound scans: Result from the witness case.

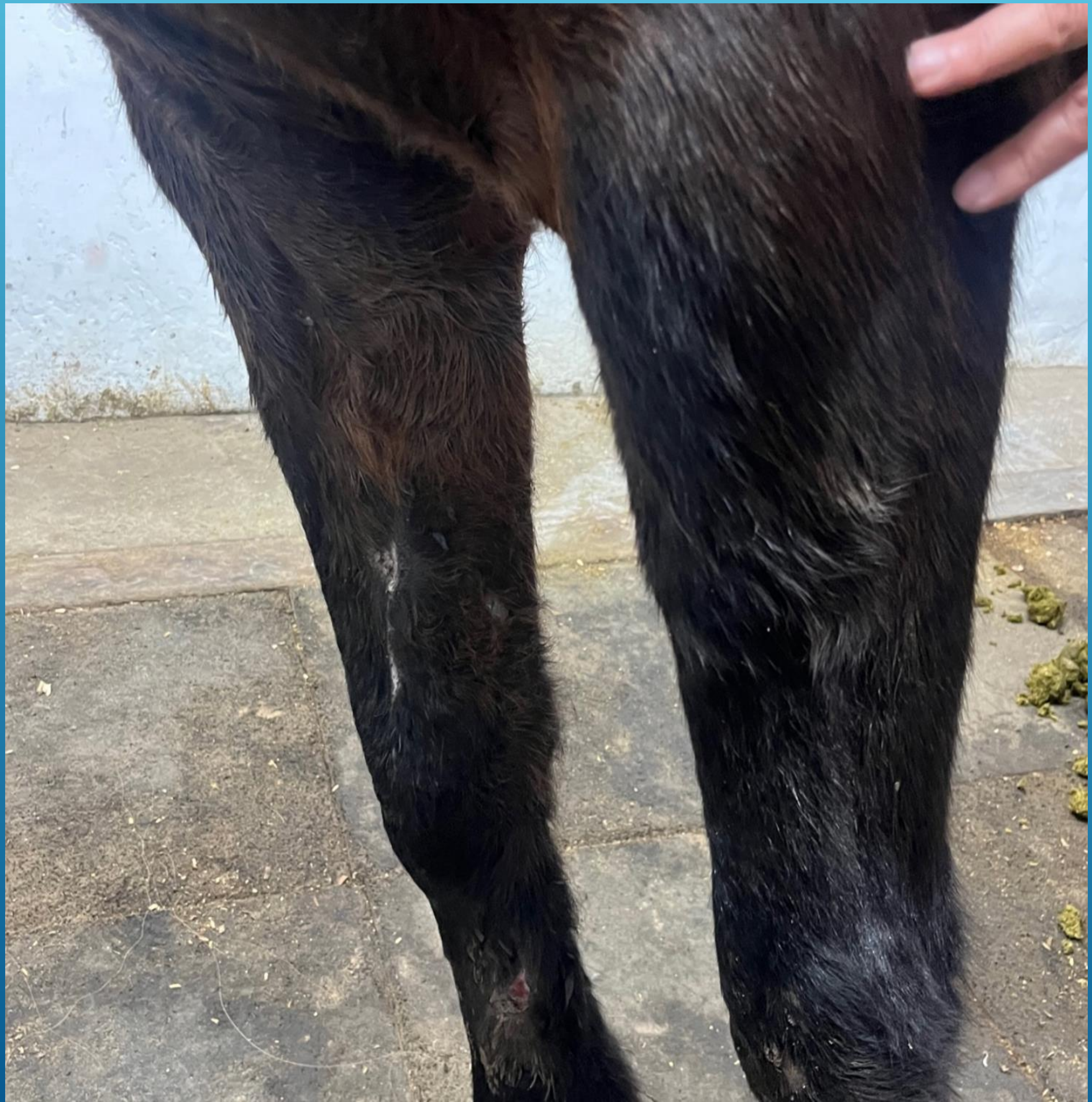

Soft tissue ultrasound was performed in the region of interest, on the site of the superficial scar. No signs of acute muscle injury are recognized at the present examan, nor are there areas of muscle fibrosis.
Consolidated skin scar is identified.

US equipement : SONOSITE M TURBO – LINEAL PROBE

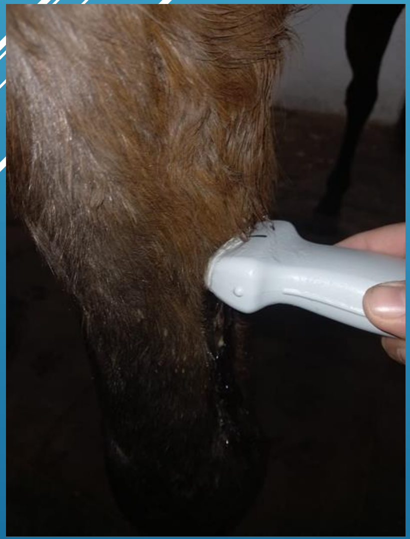

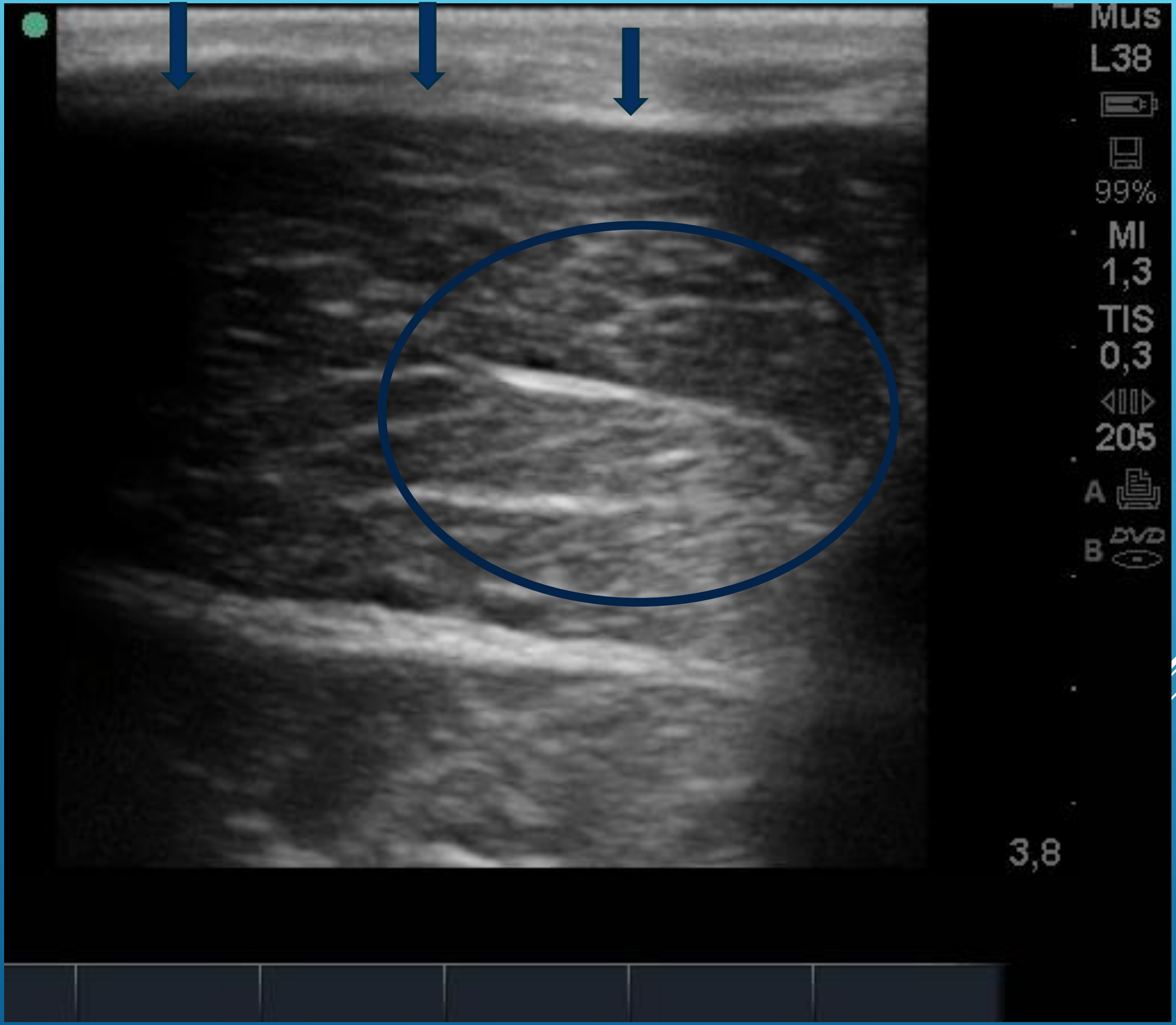

Superficial Mio-tendinous (mio-aphoneurotic) junction

Intra-muscular Mio-tendinous junction

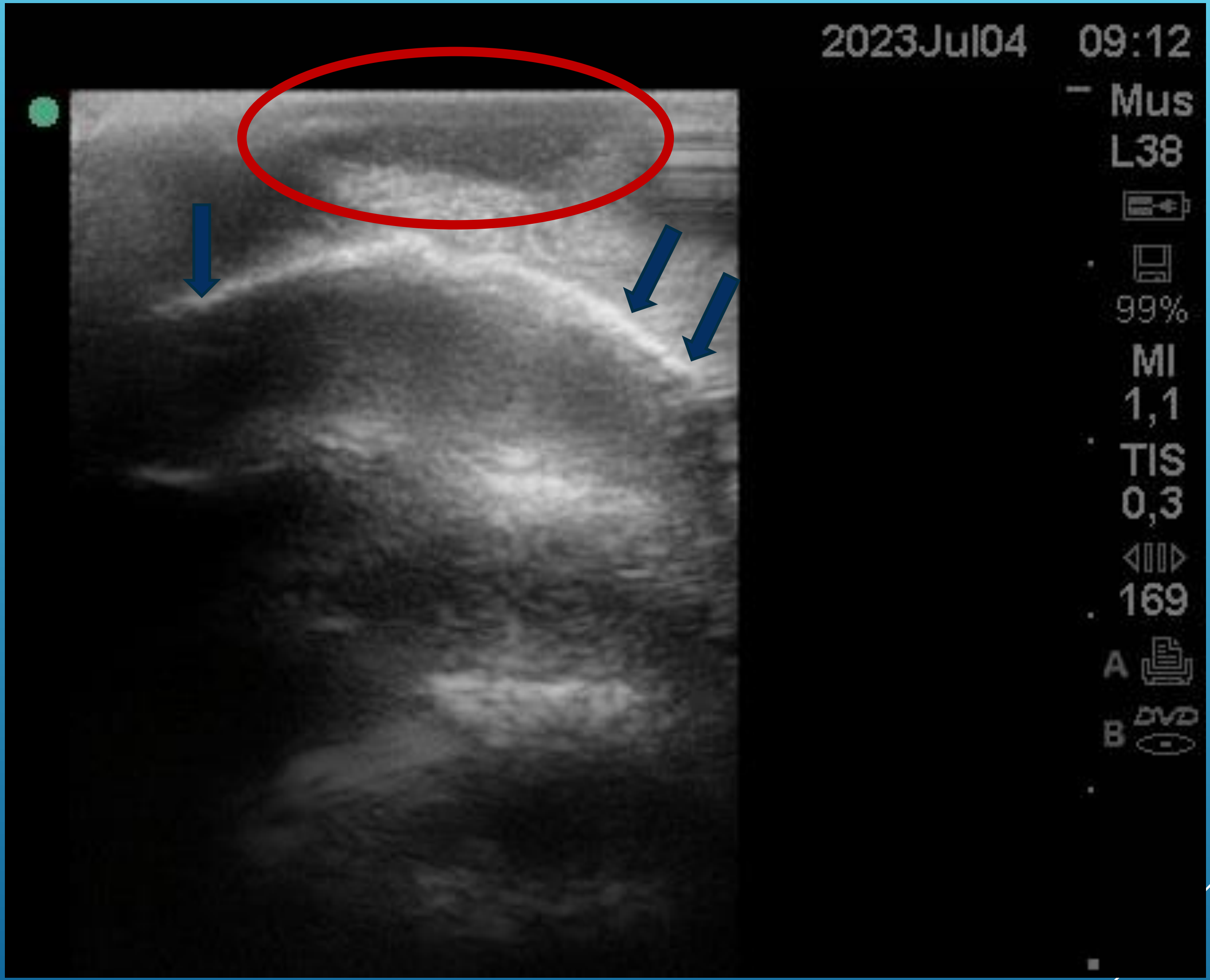

Cortical Bone

Superficial Scar

Table S1: Blood test of witness case.

(The data are given in Spanish because the original results presented by the veterinary clinical analysis laboratory have been transcribed.

HEMOGRAMA

RECUENTO CELULAR (Unidades)		Valor de Ref.
Hematíes	10,2 millon./mm3	8,5 a 10,5
Leucocitos	11,3 mil/mm3	5,5 a 10,5
Hematocrito	45 %	39 a 45
Hemoglobina	16,3 g./dl	13,0 a 17,0
**IND. HEMATIMÉTRICOS**		
VCM	44 fl.	42,5 a 46
HbCM	15,9 pg	15,3 a 16,3
CHbCM	36,2 %	33,3 a 37,8
**FORM. LEUC. RELATIVA**		
Neutròfilos en cayado	%	
Neutròfilos segmentados	55 %	46 a 60
Eosinòfilos	1 %	1 a 5
Basòfilos	0 %	0
Linfocitos	43 %	30 a 45
Monocitos	1 %	2 a 6

✓ **Fecha: 19/4/2023**

HEMOGRAMA

RECUENTO CELULAR (Unidades)	
Hematíes	9,8 millon./mm
Leucocitos	9 mil/mm3
Hematocrito	43 %
Hemoglobina	16,6 g./dl
**IND. HEMATIMÉTRICOS**	
VCM	43 fl.
HbCM	15 pg
CHbCM	36 %
**FORM. LEUC. RELATIVA**	
Neutròfilos en cayado	%
Neutròfilos segmentados	53 %
Eosinòfilos	2 %
Basòfilos	0 %
Linfocitos	43 %
Monocitos	2 %

✓ **Fecha: 3/5/2023**

HEMOGRAMA

RECUENTO CELULAR (Unidades) .	
Hematíes	9,9 millon./mm
Leucocitos	8,1 mil/mm3
Hematocrito	44 %
Hemoglobina	16,8 g./dl
**IND. HEMATIMÉTRICOS**	
VCM	44 fl.
HbCM	14 pg
CHbCM	35 %
**FORM. LEUC. RELATIVA**	
Neutròfilos en cayado	%
Neutròfilos segmentados	55 %
Eosinòfilos	1 %
Basòfilos	0 %
Linfocitos	43 %
Monocitos	1 %

✓ **Fecha: 15/5/2023**
